# Towards Marker-Assisted Breeding for Black Rot Bunch Resistance: Identification of a Major QTL in the Grapevine Cultivar ‘Merzling’

**DOI:** 10.3390/ijms24043568

**Published:** 2023-02-10

**Authors:** Paola Bettinelli, Daniela Nicolini, Laura Costantini, Marco Stefanini, Ludger Hausmann, Silvia Vezzulli

**Affiliations:** 1 Center Agriculture Food Environment (C3A), University of Trento, 38098 San Michele all’Adige, TN, Italy; 2 Grapevine Genetics and Breeding Unit, Research and Innovation Centre, Fondazione Edmund Mach, 38098 San Michele all’Adige, TN, Italy; 3 JKI Institute for Grapevine Breeding, Geilweilerhof, 76833 Siebeldingen, Germany

**Keywords:** ascomycetes, linkage mapping, phenotyping, *Phyllosticta ampelicida*, plant disease, *Vitis*

## Abstract

Black rot (BR), caused by *Guignardia bidwellii*, is an emergent fungal disease threatening viticulture and affecting several mildew-tolerant varieties. However, its genetic bases are not fully dissected yet. For this purpose, a segregating population derived from the cross ‘Merzling’ (hybrid, resistant) × ‘Teroldego’ (*V. vinifera*, susceptible) was evaluated for BR resistance at the shoot and bunch level. The progeny was genotyped with the GrapeReSeq Illumina 20K SNPchip, and 7175 SNPs were combined with 194 SSRs to generate a high-density linkage map of 1677 cM. The QTL analysis based on shoot trials confirmed the previously identified *Resistance to Guignardia bidwellii* (*Rgb*)1 locus on chromosome 14, which explained up to 29.2% of the phenotypic variance, reducing the genomic interval from 2.4 to 0.7 Mb. Upstream of *Rgb*1, this study revealed a new QTL explaining up to 79.9% of the variance for bunch resistance, designated *Rgb*3. The physical region encompassing the two QTLs does not underlie annotated resistance (*R*)-genes. The *Rgb*1 locus resulted enriched in genes belonging to phloem dynamics and mitochondrial proton transfer, while *Rgb*3 presented a cluster of pathogenesis-related Germin-like protein genes, promoters of the programmed cell death. These outcomes suggest a strong involvement of mitochondrial oxidative burst and phloem occlusion in BR resistance mechanisms and provide new molecular tools for grapevine marker-assisted breeding.

## 1. Introduction

Grapevine is one of the most renowned fruit crops in the world. Europe grows the highest proportion of grapes (50%), followed by Asia (23%), the Americas (20%), Africa (5%), and Oceania (2%) [1]. Besides the making of noble products, such as wine, table grapes, and raisins, grapevine provides transformed products, such as juices, jams, and jellies of local interest, as well as wine industry by-products, such as must, marc distillates, marc pulp, tartaric acid, seed oil, and vinegar. All these commodities follow a growing trend and include grapevine among the multi-purpose crops. Most of the grapes produced worldwide are from cultivars of the Eurasian *Vitis vinifera* L. subsp. *vinifera*, while the rest are from interspecific hybrids with other American and East/Central Asian *Vitis* species [2,3].

Regrettably, the high susceptibility of *vinifera* varieties to most fungal diseases, such as downy mildew, gray mold, and powdery mildew [4], leads to the intensive use of fungicides in viticulture. To reduce the usage of chemicals, a potential solution that is being gradually adopted is offered by the development of novel cultivars resistant to the main pathogenic threats. Actually, genetic improvement for biotic stress resistance is a valuable strategy to embrace the principles of the European Green Deal—which will be one of the strongest drivers in the Agrifood sector—aiming at the goal of 50% pesticide reduction by 2030 [5]. In fact, the crossbreeding approach has contributed to substantial changes in defense regimes, but it also resulted in the emergence of secondary diseases, which were previously controlled [6]. In fact, it has been reported that the cultivation of new varieties resistant to downy and powdery mildew—whose management needs less copper and sulfur-based treatments—has favored the spread of black rot (BR) [7]. The causal agent of BR, *Guignardia bidwellii* (Ellis) Viala & Ravaz, is a hemibiotrophic ascomycete [asexual morph *Phyllosticta ampelicida* (Engelm.) Aa]. Black rot can attack all the green expanding organs of the plant, with young shoots (leaves and internodes) and fruits being extremely sensitive. The infection is divided into an initial biotrophic symptomless phase and a subsequent necrotrophic and damaging phase [8]. On leaves, the first occurrence is the appearance of circular lesions on the adaxial surface that evolve into light brown to reddish, with darker borders. Then, the central portion turns necrotic, and the fruiting bodies (pycnidia) arise as small black dots. On the fruits, the fungus causes the formation of small whitish circles that expand concentrically around the berry, forming a brown patch. Later, the pycnidia develop as the berries rot and shrink, turning into the so-called “black mummies” [9].

Given its cultural and economic importance, the grapevine has received increasing attention from the scientific community in the last two decades, resulting in considerable progress in genetics/genomics research, as well as in phenomics approaches [10]. However, despite the intense work that has been done to study the life cycle of the BR pathogen [11,12,13,14,15,16,17,18,19,20], the genetic basis of the resistance to the disease has not yet been fully dissected. In fact, 31 and 14 resistance genetic loci—the quantitative trait loci (QTL)—have been identified so far in association with downy and powdery mildew, respectively. Instead, only two resistance loci are known for BR [21], which have been identified in the resistant donor Börner (*V. riparia* Gm183 × *V. cinerea* Arnold). To this purpose, phenotyping methods have recently been optimized in view of large-scale experiments to evaluate BR resistance on growing shoots in the greenhouse [22]. On the contrary, BR resistance of bunches has been evaluated mainly in the field upon natural infection [23,24], while only three studies report artificial inoculation of the fruit [12,16,25]. Fruits are characterized by significant ontogenic resistance [16], with a susceptibility window from six until ten weeks after bloom, depending on the cultivar [26]. Bunch resistance has been assessed only sporadically for QTL analysis [27,28,29], even if its disease tolerance should be of primary importance, since the fruit is the most valuable part of the plant. 

The present research aimed to explore the genetic basis of the grapevine BR resistance at the leaf, shoot internode, and bunch level in a complex hybrid to broaden our knowledge about the resistance mechanisms and to provide new molecular tools for genetic improvement.

## 2. Results

### 2.1. Phenotyping

**Figure 1:**
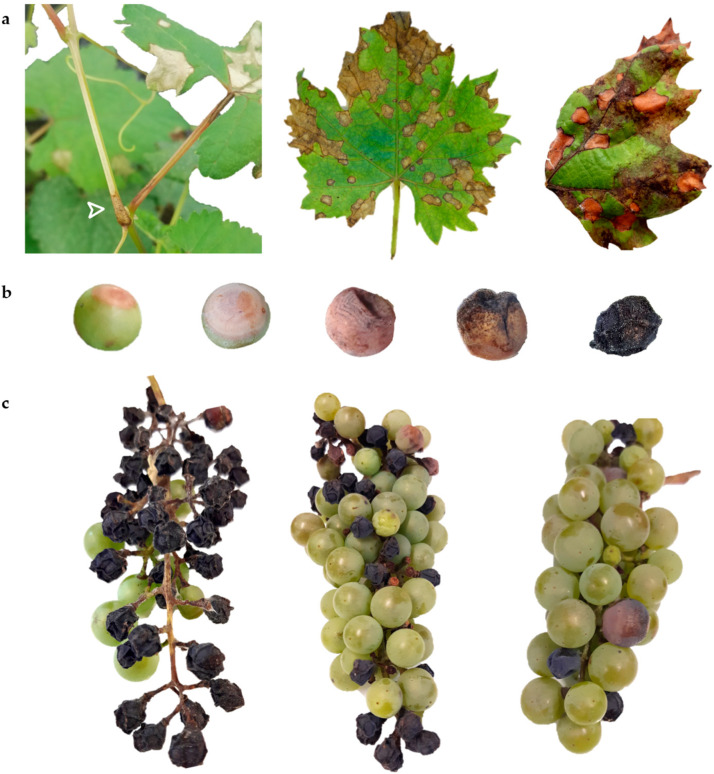
Black rot symptoms on different organs. (**a**) From the left: shoot internode, greenhouse and field leaves. Foliar lesions showed the typical circular shape, with a light brown to reddish color and rounded and darker borders. In the field, the co-occurrence of downy mildew (dark brown lesions) was evident. (**b**) Disease progression on the berry: after the appearance of a small dot, the lesion expanded concentrically, rapidly producing pycnidia; later, the berry dried up, turning into black mummy. (**c**) Clusters showing different degrees of resistance, from the left: very low (1), medium (5), high (7) (for resistance descriptors see M&M 4.2).

**Figure 2:**
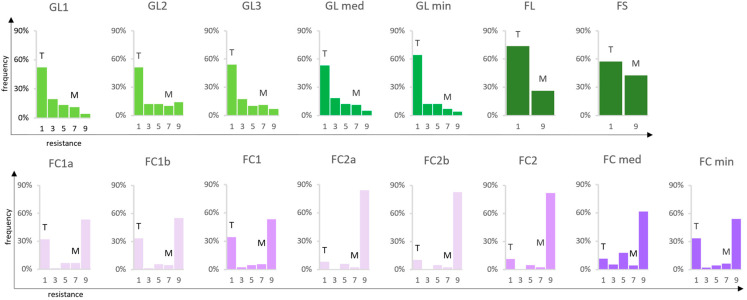
Phenotypic distribution within the M × T population (n = 147). The black rot resistance trait did not follow a normal distribution in any of the experiments (Shapiro–Wilk *p* < 0.05). Both greenhouse (GL1, GL2, GL3, GL median and minimum) and field (FL, FS) shoot evaluations were skewed towards susceptibility, while cluster resistance trials (FC1, FC2, FC median and minimum) and sub-experiments (FC1a and b, FC2a and b) were skewed towards resistance. On the *y* axis the frequency of F1 individuals is shown, while the *x* axis indicates the degree of resistance: 1 = very low; 3 = low; 5 = medium; 7 = high; 9 = very high (for resistance descriptors see M&M 4.2). Abbreviations: FC = field cluster; FL = field leaf; FS = field shoot internode; GL = greenhouse leaf; M = ‘Merzling’; T = ‘Teroldego’.

Resistance to the BR causal organism *P. ampelicida* was evaluated screening a segregating population derived from the resistant complex hybrid ‘Merzling’ (M, *V. aestivalis* var. *lincecumii* × *V. rupestris* × *V. vinifera*) and the susceptible cultivar ‘Teroldego’ (T, *V. vinifera*) (M × T, F1 individuals = 147). Overall, leaf resistance was evaluated in the greenhouse in three independent experiments (greenhouse leaves, GL1, GL2, and GL3), and once in the field (field leaves, FL), along with shoot internode resistance (field shoot internode, FS) field inoculation in 2021. In the untreated vineyard, inoculated leaves presented also downy mildew lesions (Figure 1a). Two experiments were conducted on bunches (or clusters) in the two growing seasons, 2020 (field clusters, FC1) and 2021 (FC2). In each FC inoculation trial, the entire population was screened in duplicate (FC1a and b and FC2a and b). The evolution of BR lesions on the berry and symptomatic bunches is shown in Figure 1b,c. A five-step resistance rating scheme was used, i.e., (1) very low, (3) low, (5) medium, (7) high, and (9) very high (for resistance descriptors see M&M 4.2). Under the high pressure of the greenhouse experiments, disease symptoms were visible after 14 days post-inoculation (dpi), while under natural field conditions, disease progression took longer (up to 21 dpi). The performance of the parents was stable, with ‘Teroldego’ always resulting fully susceptible and ‘Merzling’ highly resistant, and the phenotypic distribution was non-normal in all experiments (Shapiro–Wilk *p* < 0.05): for leaves and shoot internodes it was skewed towards susceptibility, while for bunches towards resistance (Figure 2). All phenotypic data are reported in Table S1.

### 2.2. Genotyping and Linkage Mapping

The M × T segregating population was genotyped by means of the GrapeReSeq Illumina 20K SNPchip. The sample of one F1 individual with a call rate of 66.5% was excluded from the dataset, which was, hance, reduced to 146 individuals. Of the 18,071 available SNPs [30], 221 were manually edited. After SNP scoring, a total of 7175 robust SNPs (39.7%) were retained and 10,898 (60.3%) discarded, of which the majority—9312, that is 51.5% of the total number of SNPs—were monomorphic. 

An integrated map and the related parental maps of ‘Merzling’ and ‘Teroldego’ were constructed with the entire marker dataset of 7175 SNPs and 194 SSRs (whole integrated map, wIN map), producing 19 linkage groups (LGs) with a minimum grouping logarithm of the odds (LOD) of 14. After excluding poorly performing markers, the wIN map presented 7358 markers, of which 3249 were unique, i.e., with no identical segregation pattern, and used to produce reduced (rIN, rM, and rT) maps with only non-redundant markers (Table 1). Segregation patterns were coded following the JoinMap^®^ segregation classification [31]. The complete and reduced datasets differed predominantly in the number of biallelic markers heterozygous only in one parent. In fact, the number of *lm* × *ll* and *nn* × *np* markers were reduced to a third, *hk* × *hk* resulted in a reduction of only a sixth, and the SSRs with segregation type *ab* × *cd* and *ef* × *eg* were all retained in the reduced dataset (Table S2). The description of the maps at single LG level is available in Table S3. 

**Figure 3:**
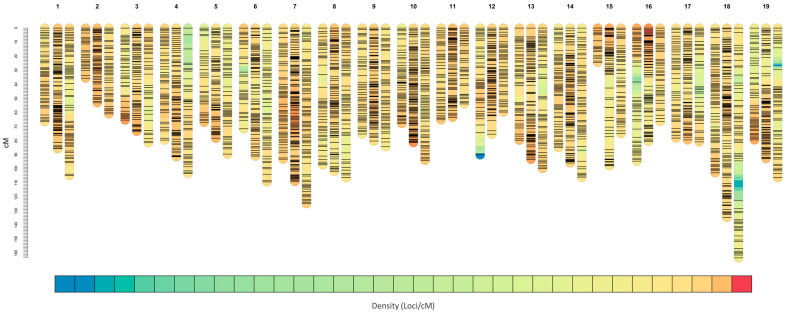
Marker density plot of the genetic maps of the M × T population. Each linkage group (LG) of the integrated map is labeled with successive numbers and positioned between the correspondent ‘Merzling’ (left) and ‘Teroldego’ (right) LGs. Marker density is represented by a color scale from blue (lowest) to red (highest). Rendering adapted from LinkageMapView [32].

The unique bins (loci) of the IN map were 1613, with an average inter-locus gap distance of 1.04 centimorgan (cM) and a map density of 0.96 bins/cM, while the T parental map presented a lower density of 0.56 bins/cM, with the higher inter-locus gap of 1.80 cM, due to the reduced number of loci per map unit (Table 1, Figure 3). Instead, the M parental map resulted the shortest, with 1452 cM and a density of 0.58 bins/cM. The lack of polymorphic markers in some regions determined the presence of major gaps in the maps. The integrated map presented the biggest gap of 9.5 cM on LG15. In the M map, LG12 showed a big lack of markers at the distal arm, with a gap of 15.7 cM. On LG18, the T map presented the biggest gap among all maps, of about 28 cM. Since the grouping of the integrated map is based on the recombination of alleles in the progeny, linked markers in the integrated map may not be linked in the parental ones. This occurred for LG15 in the M map, where the lack of polymorphism did not allow for linking the two portions of the linkage group (the distal one having only two bins), producing a shorter—and the shortest—linkage group of about 24 cM (Figure 3). A detailed correspondence between the whole integrated and parental maps is depicted in Figure S1. A higher quality of doubled-haploid (dh) maps were obtained using the Regression mapping algorithm, with Kosambi’s instead of Haldane’s mapping function, suggesting crossover interference. The dhM map (Table S3, Figure S2) was generated based on a minimum grouping LOD of 14; it measured 943 cM and comprised 998 unique markers, sorted in 791 loci, for an average inter-locus distance of 1.19 cM. As expected, the distal branch of LG15 grouped separately prematurely (LOD 3), determining the minimum value of 9 bins observed within all the developed maps. The major gap of 13.2 cM was present on LG6. The dhT map (Table S3, Figure S3) was based on a minimum grouping LOD of 8; it resulted 1346 cM long, counting 1137 markers and 917 unique bins, which determined a higher mean distance of 1.47 cM between loci.

### 2.3. Quantitative Trait Locus Analysis

Due to the non-normal distribution of the phenotypic traits (Figure 2), Kruskal–Wallis (KW) non-parametric analysis was applied on the wIN map to highlight the most strongly associated markers. The analyses with GL and FC overall minimum and median values gave the same results within each experiment type; therefore, only GL min and FC min values were included in the following analysis. 

**Figure 4:**
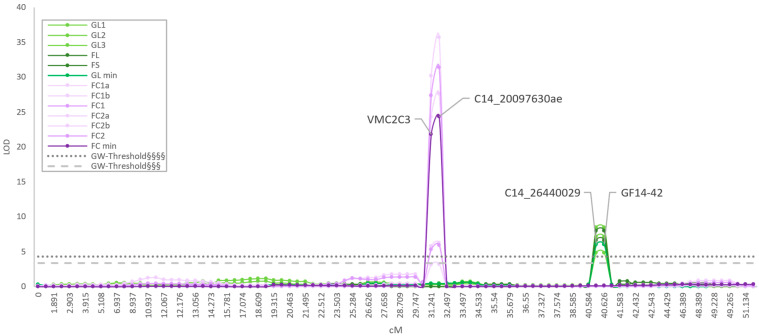
Results of MQM analyses on LG14. A clear separation between bunch (purple) and shoot (green) resistance is evident. The associated markers are reported. Significance is represented by GW^§§§§^ (*p* < 0.01) and GW^§§§^ (*p* < 0.05) thresholds. On the *y* axis the LOD value is shown, while the *x* axis marker position in cM. Abbreviations: cM = centimorgan; FC = field cluster; FL = field leaf; FS = field shoot internode; GL = greenhouse leaf; GW = genome-wise; LG = linkage group; LOD = logarithm of odds; MQM = Multiple QTL Mapping. Official names are shown for SSR markers, for the original names of SNP markers see Table S11.

To define resistance QTLs, Multiple QTL Mapping (MQM) analyses were performed on the resistant parent dhM map. The segregation of the berry color trait worked fine as a positive control for the QTL analyses, identifying the proper position of the locus BeCo [33]. All BR experimental datasets revealed only one single QTL on LG14. The significance always exceeded the most stringent *p* < 0.01 (^§§§§^) genome-wise (GW)-threshold (Table S4), but with a different peak position for the experiments on shoot (leaf and shoot internode) compared to bunches (Figure 4, Table 2), and a minimum phenotypic variance explained (PVE) of 15.7%, hence making all of these major QTLs [34].

The QTL associated with shoot resistance was located between 40.584 and 40.626 cM (two-LOD support interval). The associated marker GF14-42 (40.626 cM) explained from a minimum of 20.4% (GL2) to a maximum of 29.2% (GL1) of the phenotypic variability, and it coincided with the marker segregating with *Resistance to Guignardia bidwellii* (*Rgb)*1 locus [21]. The second marker C14_26440029 (40.584 cM) segregated with the QTL with the same LOD and PVE as GF14-42 (Figure 4, Table 2). 

The berry resistance QTL was located between 31.241 and 31.533 cM. The most significantly associated marker was C14_20097630ae, explaining from 20.3% (FC1) up to 79.9% (FC2) of the variability of the trait. A second marker co-segregated with the QTL, with the PVE ranging from 18.4% (FC1) to 75.4% (FC2). The QTL was also confirmed by analyzing the phenotypic distribution of the four FC sub-experiments (FC1a, FC1b, FC2a, and FC2b). According to previous nomenclature [21], the locus was named *Rgb*3. The KW analyses showed the highest significance (*p* < 0.000005) for all the associated markers to both loci. 

**Figure 5:**
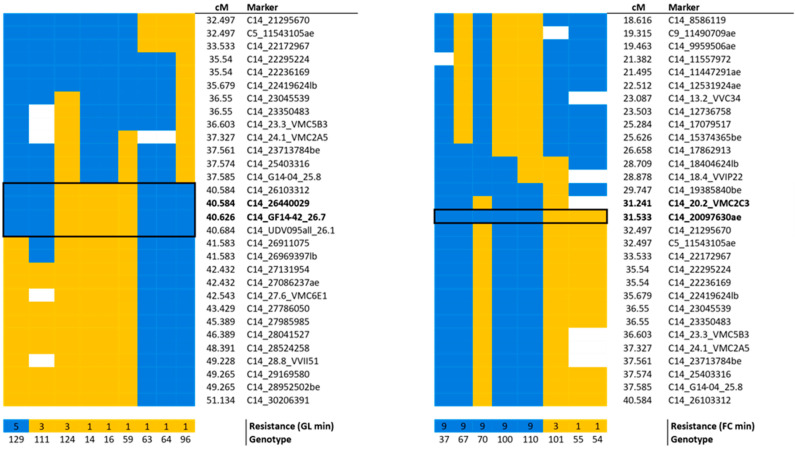
Recombinant F1 individuals at *Rgb*1 (left) and *Rgb*3 (right) loci within the dhM map. The haplotype phases are shown in orange and blue, and the color code is used to give a putative association with the overall minimum resistance level of GL and FC experiments. Missing genotypic data are shown in white. The black boxes define the minimal recombinant region, and co-segregating markers significantly associated with the QTLs are highlighted in bold. For the official marker names see Table S11, for resistance descriptors see M&M 4.2. Abbreviations: dhM = doubled haploid; FC = field cluster; GL = greenhouse leaves.

To deepen the genetic basis of BR resistance, recombinant progeny individuals at the locus were studied. Eight individuals were recombinant at *Rgb*3 locus, clearly separating the resistant (blue) and the susceptible (orange) haplotype phase (Figure 5). Instead, this approach failed to reduce the *Rgb*1 locus, since recombinant individuals defined a wider region compared to the MQM QTL analysis, comprised between 40.584 and 40.684 cM.

### 2.4. Candidate Genes

**Figure 6:**
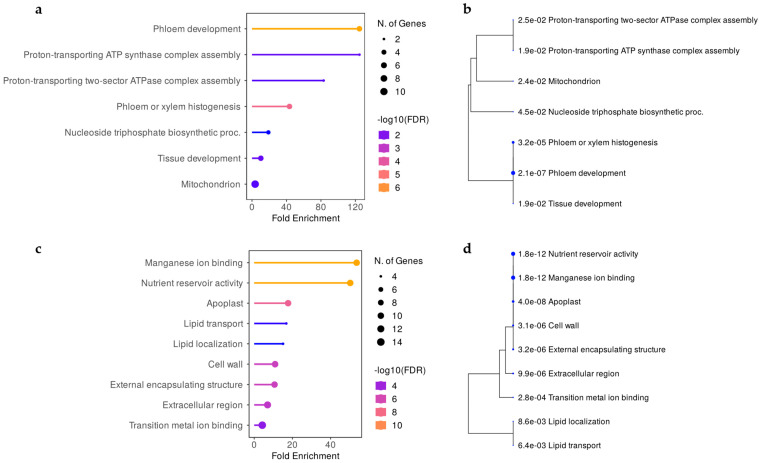
Gene Ontology (GO) enrichment and hierarchical clustering trees summarize the correlation among significant gene sets for *Rgb*1 (panel **a**,**b**) and *Rgb*3 (panel **c**,**d**) loci, respectively. Enriched categories (panel **a**,**b**) are colored based on false discovery rate (FDR, −log10 transformed). On the left, bigger dots indicate increased number of genes, while on the right side they indicate more significant *p*-values.

The QTL region associated with the BR resistance of shoot (FL, FS, and GL) colocalized with the *Rgb*1 locus and co-segregated with the same GF14-42 marker [21]. To define the physical position of the QTL, the latest version 4 of the genome assembly from the PN40024 accession (PN40024.v4) [35] was used. The alignment of the highly dense map developed in this work to this version of the grapevine reference genome allowed a size reduction of the locus from 2.4 Mb [21] to a region of 0.7 Mb, comprised between 26.6 and 27.3 Mb on chromosome 14. A total of 22 genes have been annotated within the *Rgb*1 two-LOD support interval. The associated SNP marker C14_26440029 locates within the coding sequence of the Vitvi14g01631 gene predicting for an uncharacterized membrane protein. The expanded region to the nearest non-associated flanking markers comprehended 103 genes (Table S5). The enriched categories of the *Rgb*1 locus based on gene ontology (GO) analysis of the 82 genes having GO description, and the corresponding gene lists, are available in Table S6. Three out of seven enriched gene sets were associated with phloem development (Figure 6b) and comprehended a gene cluster encoding for five unknown proteins (Vitvi14g01641, Vitvi14g01642, Vitvi14g01646, Vitvi14g02997, and Vitvi14g01653), with a fold enrichment (FE) from 10.3 to 124.7 (Figure 6a). For all of them, the best Arabidopsis match was the sieve element occlusion B (AT3G01680) (Table S5), a scaffold protein required to form the phloem filament matrix in sieve elements. Three additional enriched classes (Figure 6b) (namely ‘Proton-transporting ATP synthase complex assembly’, ‘Proton-transporting two-sector ATPase complex assembly’, and ‘Mitochondrion’) were all related to the mitochondrion (FE from 3.6 to 124.7, Figure 6a). The ATP synthase mitochondrial F1 complex assembly factor 2 (Vitvi14g01626) and Ku70-binding (Vitvi14g01636) genes were present in all three classes, together with a Pentatricopeptide (*PPR*) repeat-containing protein gene (Vitvi14g01632) in the class ‘Mitochondrion’ (Table S5). Lastly, another enriched category (FE 19.0, Figure 6a) was represented by the ‘Nucleoside triphosphate biosynthetic process’ (Figure 6b), composed of three genes coding for a dehydration-responsive protein (Vitvi14g01667), an ubiquinol-cytochrome c reductase cytochrome c1 (Vitvi14g01592), and a thymidylate kinase (Vitvi14g01593) (Table S5).

The *Rgb*3 locus was located within 19.8 and 19.9 Mb on chromosome 14, defining a region of 0.2 Mb that comprises 8 functionally annotated genes (Table S7). The most significantly associated marker, C14_20097630ae, was detected within an intron of the gene Vitvi14g01119, coding for a predicted phosphatidic acid phosphatase alpha (PAPα). The other highly associated marker, VMC2C3, was instead located within an intron of the gene Vitvi14g01125, coding for a HEAT repeat-containing protein. Two functionally annotated genes, Vitvi14g01120 and Vitvi14g01124, were predicted as an ATP binding protein and a plastid movement-impaired 15 (PMI15) protein, respectively. PMI15 is required for the so-called “avoidance response” that relocates chloroplasts on the anticlinal side of the cells in response to high-intensity blue light. The expanded region to the flanking neighboring markers (19.7 to 22.1 Mb) comprehends a list of 106 genes. No classical resistance (R)-gene was present, but, of the 66 genes having GO description, a significant enrichment (FE from 4.2 to 53.6) of genes predicted as germin-like protein 3 (Glp3) was detected. In fact, a cluster of 10 *Glp3* was present in the VCost.v3 (V3) annotation [36], and it resulted in belonging to seven of the nine enriched gene sets (Figure 6d, Table S8). The enriched ‘Extracellular region’ category, beyond the 10 *Glp3* genes, also contains two Non-specific lipid-transfer protein 2 (LTP 2) genes (Vitvi14g02869 and Vitvi14g01128), while the enriched ‘Transition metal ion binding’ category counted additional four genes that encoded, respectively, a delta 7-sterol-C5-desaturase (Vitvi14g01152), a mannose-6-phosphate isomerase (Vitvi14g01178), a cytokinin hydroxylase (CYP735A1, Vitvi14g01195), and a DNA-directed RNA polymerase subunit beta’ (Vitvi14g01068). (Figure 6c, Table S7). Finally, other two enriched pathways were linked to lipid transport and localization (FE 16.8 and 15.1), and comprehended the same four genes that predict a HEAT repeat-containing protein (Vitvi14g01125), two non-specific lipid-transfer protein 2 (Vitvi14g02869, Vitvi14g01128), and a synaptotagmin (Vitvi14g01186).

**Figure 7:**
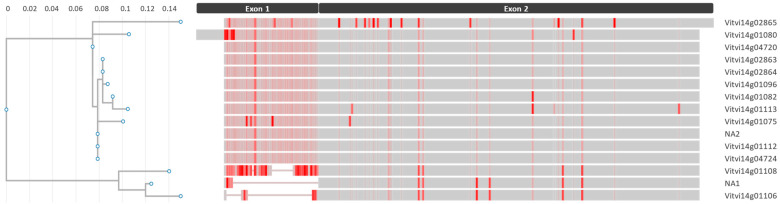
Phylogram (left) of *Glp3* gene cluster at *Rgb*3 locus and multiple alignment visualization (right) highlighting protein sequence conservation. Gaps in the alignment are represented as lines, darker shades of red indicate a greater difference than the consensus at that position, while gray represents 100% consensus. The branching, of the phylogram is based on SH-like local supports (tick values) that measure the degree of genetic change ranging from 0 to 1.

By blasting the sequence of Vitvi14g01075 *Glp3* on PN40024.v4, five more genes were identified within the cluster, bringing the total number of genes to 15 (Figure S4), whose structural annotation, hence, underwent manual curation. In two cases (Vitvi14g01106 and Vitvi14g02865, V3 annotation), the genes erroneously unify two open reading frames (ORFs). These were split, resulting in two new genes annotated in the PN40024 version 4.3 (V4.3) [37] as Vitvi14g04724 and Vitvi14g04720, which corresponded to VIT_14s0006g02400 and VIT_14s0006g02440 in the CRIBI version 1 annotation (V1) [38]. In addition, two new genes were annotated starting at position 19,604,399 (gene name nota available 1, NA1) and 19,612,558 (gene name NA2), which are in progress to be approved by the curators of PN40024.v4. The manual curation was supported only by the non-PN40024 RNAseq evidence, except for one gene (Vitvi14g01096) supported by PN40024 RNA-Seq data. Instead, four ORFs showed no supporting expression data (Table S9). The multiple alignment of the amino acid (aa) sequences revealed a very high mean identity of 94.3%, and the three sequences NA2, Vitvi14g01112, and Vitvi14g04724 and the two sequences Vitvi14g02863 and Vitvi14g02864 were identical within each other (Figure 7 and Figure S4). Conversely, the lowest identity value (79.5%) was obtained by the pairwise alignment between Vitvi14g01108 and Vitvi14g02865 (Figure S4). The alignment showed a greater identity of the second exon, while the region of increased variability was in the first exon. In particular, 10 genes shared a predicted protein length of 221 aa, while the genes NA1, Vitvi14g01106, and Vitvi14g01108 showed gaps in the first exon, and the genes Vitvi14g01080 presented an extra upstream sequence (13 aa), and the gene Vitvi14g02865 an extra downstream (7 aa) sequence (Figure 7 and Figure S4).

**Figure 8:**
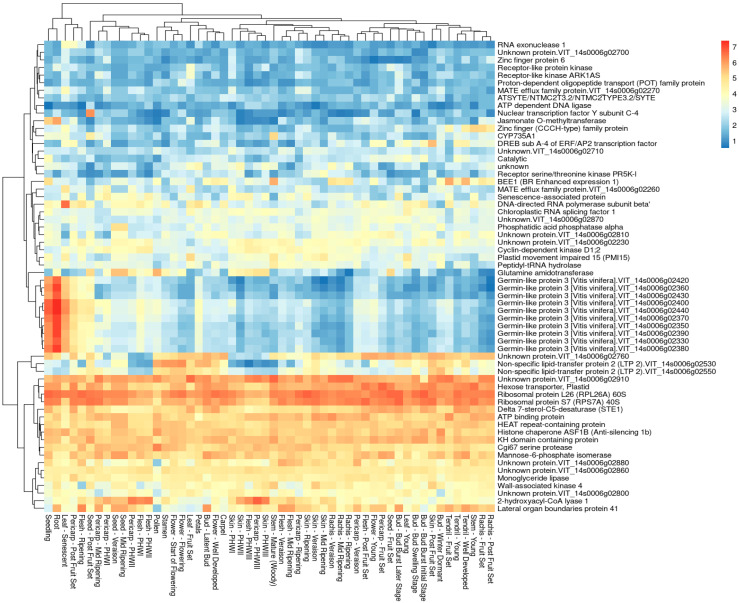
Transcriptomic analysis of *Rgb*3 locus genes in different tissues and developmental stages using the VITVIZ ‘Corvina’ ATLAS [39]. Blue and red colors represent low and high expression levels, respectively (logarithmic data transformation).

Finally, publicly available data [39] were exploited to investigate the expression pattern of the genes belonging to the *Rgb*1 and *Rgb*3 loci on chromosome 14. The produced heat-maps showed that the clustered *Glp3* genes on chromosome 14 are highly expressed in roots, seedlings, post fruit set pericarp, and senescent leaves (Figure 8), with the last three developmental stages known to be recalcitrant to BR infection. Contrarywise, the genes belonging to the *Rgb*1 locus did not show specific patterns (Figure S5).

## 3. Discussion

### 3.1. High-Density SNP Coupled with SSR Genotyping Provides Highly Informative Linkage Maps

**Figure 9:**
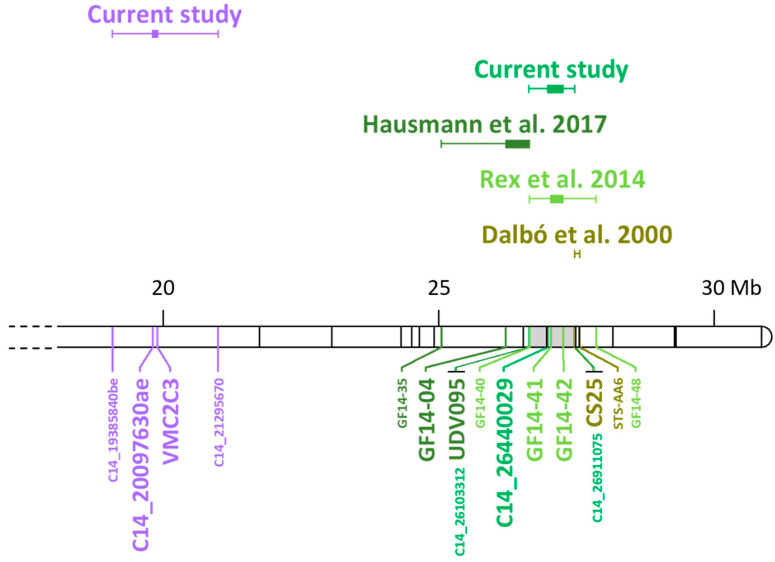
Overview of the black rot resistance QTLs at chromosome 14. The map is an integration between the current and the previous [21,40,42] studies, based on marker position (Mb) on PN40024.v4 [35]. The box plots represent QTL intervals based on co-segregating markers (larger font) expanded (error bars) to the nearest non-associated flanking markers (smaller font). The *Rgb*3 associated to bunch resistance detected in the current study is depicted in purple. The *Rgb*1 locus associated with leaf resistance in the current and previous works [21,40,42] are colored with different shades of green. The same color code is given for the respective markers. The overlap of the analyses is highlighted in grey. Official names are shown for SSR markers, for the original names of SNP markers see Table S11.

The construction of a map combining existing SSR to new SNP information was an effective strategy that eased the comparison of the results obtained in previous works. In fact, in this study, we were able to confirm the co-segregation of BR shoot resistance in the grapevine cultivar ‘Merzling’ (*V. aestivalis* var. *lincecumii × V. rupestris × V. vinifera*) with the SSR marker GF14-42, previously described by Rex et al. [21] as associated with the *Rgb*1 locus mapped on LG14 in the rootstock variety ‘Börner’ (*V. riparia × V. cinerea*) (Figure 9). Moreover, the developed highly dense map allowed for narrowing down the size of the QTL to 0.7 Mb. The same region has been identified also by Hausmann et al. [40] in a breeding population with a complex inter-specific background (*V. aestivalis* var. *lincecumii × V. cinerea* var. *berlandieri × V. labrusca × V. rupestris × V. vinifera*). In that work, the locus has been linked to the SSR marker UDV-095 [41], which was included in the current marker set and colocalized with GF14-42 in the M map (Figure 9). Finally, Dalbó et al. [42] identified the same genomic region associated with both BR and the powdery mildew resistance locus *Resistance to Erysiphe necator* (*Ren*)2 in the maternal map of the population ‘Illinois 547-1’ (*V. cinerea* B9 *× V. rupestris* B38) × ‘Horizon’ (*V. aestivalis* var. *lincecumii × V. labrusca × V. rupestris × V. vinifera*). However, the resistance-linked RAPD marker CS25b [42] turned out to be monomorphic in the present study. Anchoring these four studies to the PN40024.v4 genome [35], an overlapping region of about 0.82 Mb emerged (Figure 9). Based on these findings and on a preliminary genotypic screening of marker allele segregation in different genetic backgrounds—which will be thoroughly investigated in a future work—it can be asserted that this region of the chromosome 14 is crucial for BR resistance, and different haplotypes could co-exist, analogously to the *Resistance to Plasmopara viticola* (*Rpv)*3 multi-haplotype locus [43]. Another advantage of including SSR markers in highly dense SNP-based maps is their informativeness and technical ease of validation in different genetic backgrounds, whereas SNP markers need to be converted into haploblocks first and then checked back first to the original mapping population. Nevertheless, a bottleneck of SNPchip genotyping is that some regions are over- or under-represented, due to the coverage and depth of previous versions of *V. vinifera* genomes. In addition, the 18K chip sequencing design dates to 2013 [30]; consequently, it lacks some *Vitis* species that nowadays have been largely employed into grapevine breeding programs, such as the American *V. arizonica*, *V. riparia*, and *V. rupestris* and the Asian *V. amurensis*, *V. piasezkii*, and *V. romanetii* listed in the ‘Table of Loci for Traits in Grapevine Relevant for Breeding and Genetics’ from *V*IVC [44]. To address this problem when working with those genetic backgrounds, genotyping-by-sequencing (GBS) is often preferred, e.g., [45,46], although GBS raises the problem of the low transferability of the outcomes (missing primer and sequence information).

Concurrently with more precise QTL mapping, the highly dense maps allowed the evaluation of recombinant individuals (Figure 5), which is a good strategy to further investigate and narrow down the genetic basis of a particular trait when it determines a wide percentage of the phenotypic variability [29]. Instead, this approach fails to define loci with complex inheritance, e.g., for the powdery mildew stem resistance locus on chromosome 8 [29] and for the *Rgb*1 locus. In this regard, possible epistatic effects of the *V. vinifera* parent ‘Teroldego’ on the resist trait have been hypothesized. The ’Teroldego’ genetic background did not influence the *Rgb*1 trait segregation, but further studies are ongoing to determine its contribution. In fact, a previous work [47] demonstrate that for the same resistance donor, the *V. vinifera* cultivar with which it is crossed can influence the expression of the trait.

### 3.2. Resistance Evaluation of Shoot and Bunch Reveals Organ-Specific QTLs

The present work corroborated the known foliar *Rgb*1 locus and reported the first genetic dissection of BR bunch resistance (*Rgb*3). This finding is a breakthrough since—despite the most valuable product of viticulture are of course grapes—in resistance assessments, mainly leaves are evaluated for experimental reasons. Analogously to other fungal diseases, the occurrence of BR infection on different organs during one season is well-established, introducing the concept of “dual epidemics” [48]. To this purpose, the development of new evaluation protocols of downy mildew resistance on detached inflorescences has been addressed to study divergent dual epidemics cases [49]. However, the outcome of ex vivo experiments in our previous study [22] highlighted the impossibility to follow BR disease progression on detached organs. Therefore, resistance assays on grape berries had to be performed in the field necessarily, followed by the development of a new descriptor for bunch resistance (Table 1). 

The detection of organ-specific QTLs has been barely described in grapevines for a very few pest and disease resistance cases, i.e., phylloxera root and foliar resistance [27,50], and multi-organ powdery mildew resistance [29]. This phenomenon is well known and has been studied through QTL mapping also, e.g., for potato late blight [51], maize common smut [52], and the two different diseases head blight (or scab) and crown rot that affect both wheat and barley and are caused by the same *Fusarium* pathogens, recently reviewed by Su et al. [53]. As a consequence, multi-organ evaluation at multi-developmental stages might be of paramount importance for BR disease since there is evidence of a positive effect of senescence on the resistance trait [16], an observation supported by experimental evidence by the expression pattern of Germin-like 3 genes underlying the *Rgb*3 locus (Figure 8).

### 3.3. The Genes Underlyng Rgb Loci Suggest non-R-Gene-Mediated Resistance Mechanisms

Following the comprehensive candidate gene analysis, no classical R-gene was present within the two identified genomic intervals. The R-genes are characterized by the highly conserved NLR (nucleotide-binding leucine-rich repeat) domains required for protein–protein interaction [54] that allow the host to activate the innate plant effector-triggered immunity (ETI) by the direct or indirect recognition of pathogen effector molecules (avirulence proteins, Avr) delivered into the plant cell to overcome the basal immunity (or pathogen-associated molecular patterns PAMP-triggered immunity, PTI) [55]. Therefore, the possible involvement of underlying genes in alternative resistance mechanisms at the shoot and bunch level was investigated.

#### 3.3.1. Shoot Rgb1 Locus

Upon GO enrichment analysis (Figure 6a,b), to our knowledge, this work suggested for the first time a relation between phloem sieve occlusion elements (SOEs) and shoot resistance to a fungal disease as BR. In fact, the *Rgb*1 locus includes an enriched cluster of five genes belonging to this family. Traditionally, phloem has been associated with sap-feeding insect resistance [56] since it is the vascular tissue where the translocation of soluble organic compounds occurs. Sieve occlusion elements are phloem proteins (called P-proteins) required for the formation of filamentous or tubular structures [56] that associate with organelles and stay firmly attached to the plasma membrane to avoid sieve elements occlusion [57], the latter being conducting cells, where the sugar transport produced during photosynthesis actually occurs [58]. These findings might explain the reason ex vivo experiments on detached leaves do not allow observing disease progression [22], since the separation from the plant impede phloem flow, thus limiting sugar availability to the pathogen. Moreover, they also support the well-known evidence of a preferential disease progression and pycnidia formation nearby the main leaf veins [8] that was also observed in the present study, a region where sugar translocation is more abundant. All five genes belonging to this enriched cluster encode for the first type of SOEs that comprehends scaffold proteins required to form the phloem filament matrix. The second type of SOEs is represented by callose, whose deposition is notoriously implicated in grapevine resistance to both downy and powdery mildew [59,60], as well as in other plant resistance mechanisms [61]. This suggests that the SOE type B detected in this work might act in an analogous manner to callose, limiting the nutrient uptake by the pathogen. 

A second enriched GO class was associated with the mitochondrion proton transfer. Like in animals, in plant systems, the mitochondria determine the hypersensitive response to stress stimuli, initiating programmed cell death (PCD) [62]. The synthesis and export of ATP and the generation of higher amounts of phosphate is necessary for the reduction of excessive amounts of reactive oxygen species (ROS) in the cytoplasm; therefore, the impairment of this mechanism causes ROS accumulation, irreversible membrane and DNA damage, and cell death [63]. Accordingly, the assembly factor 2 and Ku70-binding protein underlying the *Rgb*1 locus might negatively regulate ATP synthase mitochondrial F1 complex formation and proton transport, inducing the oxidative burst and, consequently, the PCD. A similar mechanism has been described through the silencing of a mitochondrial ATP synthase F1 subunit that caused improved resistance to *Ralstonia solanacearum* in *Nicotiana benthamiana* [64]. Within this perspective, another mitochondrial resistance mechanism might be mediated by the significantly associated *PPR* gene. In fact, their disruption has been demonstrated to be involved in the spontaneous cell death response caused by H_2_O_2_ accumulation, resulting in enhanced resistance to some fungal and bacterial pathogens in rice [65]. Finally, a possible role of nucleoside triphosphate biosynthetic processes can also be hypothesized, since the Thymidylate kinase homologous gene in cucumber was significantly overexpressed in a resistant line to Fusarium wilt [66].

#### 3.3.2. Bunch Rgb3 Locus

The most strongly associated marker with *Rgb*3 locus locates within a gene encoding for a PAPα enzyme. The latter is involved in the dephosphorylation of the phosphatidic acid, thus modulating the concentration of this important signal molecule, which is directly participating in the positive regulation of ROS accumulation [67]. A knock-down mutant in a homologous gene of Arabidopsis—a phosphatase with a central role in maintaining jasmonate and salicylic acid hormone homeostasis and defense signaling—was found to show enhanced susceptibility to necrotrophic pathogens, but did not affect the response to biotrophic microorganisms [68]. This suggests that PAPα could be counted among the susceptibility genes, and therefore, it is valuable for gene/base editing approaches. 

The GO analysis (Figure 6c,d) revealed a significantly enriched *Glp3* gene cluster. Germin-like proteins are secreted N-glycosylated peptides associated with the extracellular matrix [69], which are involved in plant growth and development [70], as well as in disease resistance through oxidative burst [71], hance in PCD induction. Together with the antimicrobial peptides (AMPs), they have been classified within the pathogenesis-related (PR) proteins, and their accumulation is a key component of the plant innate immune system, and especially of systemic acquired resistance (SAR), a mechanism of induced defense that confers long-lasting protection against a broad spectrum of microorganisms [72]. As a relevant gene cluster, BLAST analysis of the *Glp3* nucleotide sequences was not only conducted with the PN40024.v4 (*V. vinifera* subsp. *Vinifera*) genome [35], but also with the genomes of the putative ancestors (wild *Vitis* species) provided by Cantu Lab (UC Davis) [73]. This preliminary analysis revealed an expansion or shrinkage of the *Glp3* cluster, both “inter” (between), but also “intra” (within) the wild haplophased chromosomes, advising for the necessity to further investigate these genes in the resistance donor ancestors. In grapevine, *Glp*s were first reported in association with the ontogenic resistance of berries to powdery mildew [74]. Then, the expression of the *VvGLP3* gene—coding for a protein with superoxide dismutase (SOD) activity—was detected to be highly induced at *Erysiphe necator* infection sites [75]. These findings suggest focusing on the investigation of senescence in relation to disease resistance, which might uncover new, promising, broad-spectrum and basal resistance mechanisms. In rice, endogenous *OsGlp* gene silencing increased susceptibility to both sheath blight and blast [76,77]. The overexpression of *Glp* genes has also been demonstrated to be associated with increased resistance to *Verticillium dahliae* and *Fusarium oxysporum* in cotton [78] and to *Sclerotinia sclerotiorum* in tobacco [79]. Multiple disease resistance (MDR) has been inferred based on the detection of non-specific defense mechanisms and genes or QTL clusters associated with different diseases [80]. This phenomenon is well known and referred to as pleiotropy [81]. In maize, two QTLs have been identified as conferring broad-spectrum resistance to three and two diseases, respectively [80]. Finally, a gene family of 14 *Glp*s was studied in rapeseed, demonstrating that the early induction of the SOD activity of some Glp generate H_2_O_2_ and increase the resistance to the necrotrophic fungus *S. sclerotiorum* [71]. 

Another significantly enriched group of genes belonged to lipid transport and localization. Within the *Rgb*3 locus, there are two HEAT repeat-containing proteins, and a third one is located 0.3 Mb downstream of the expanded interval (Table S5). The latter gene results in being tightly associated with the recently published *Resistance to Plasmopara viticola* 29 (*Rpv*29) locus [82]. The study highlights the probable requirement of HEAT repeat proteins for basal non-host resistance, as it is the case for ILA in Arabidopsis [83]. This implies that the HEAT repeat protein has a pleiotropic effect and corroborates the hypothesis that the *Rgb*3 locus is a broad-spectrum resistance locus. The lipid transport and localization category also encompassed two non-specific lipid-transfer protein 2 (nsLTPs), considered for all intents the most abundant AMP protein family [84]. Two nsLTPs from maize have also been biotechnologically engineered to obtain peptides able to protect pearl millet from downy mildew [85]. Finally, the ‘Transition metal ion binding’ enriched gene set includes, besides *Glp3* genes, also a mannose-6-phosphate isomerase. Mannose is thought to play an important role in ascomycetes and oomycetes, but also basidiomycetes, and the impairment of its metabolism can cause a reduction in glucose, fructose, and mannose, thus generating chain-inhibitory effects based on the limitation of nutrient uptake that can block initial infection [86]. 

## 4. Materials and Methods

### 4.1. Segregating Population

An interspecific population was generated in 1998 by crossing ‘Merzling’ and ‘Teroldego’ (M × T) at the Fondazione Edmund Mach (FEM, San Michele all’Adige, Italy). Based on pedigree reconstruction [22,87], ‘Merzling’ is a complex hybrid among *V. vinifera*, *V. rupestris*, and *V. aestivalis* var. *lincecumii*, which displays high resistance against BR, while ‘Teroldego’ is a BR susceptible *V. vinifera* landrace of the Trentino region [22]. The population comprised more than 150 individuals that were planted in an untreated open field at San Michele all’Adige (Trento, Italy) as single plants. For greenhouse experiments, parental and progeny buds were collected for propagation, as described by Vezzulli et al. [88]. Excluding plants derived from self-pollination and outcrossing identified through microsatellite analysis, 147 genetically unique individuals of the M × T progeny were included in this study.

### 4.2. Phenotyping

Two strains of *P. ampelicida* isolated by FEM in Trentino (Italy), and one isolated by the Julius Kühn Institute (JKI)–Institute for Grapevine Breeding Geilweilerhof (Siebeldingen, Germany), were previously genetically characterized and propagated on oatmeal agar (0.5% *w*/*v*) [22]. These strains were combined and used for artificial infection, following the protocol developed by Bettinelli et al. [22]. Briefly, fresh leaf tissues with mature BR lesions were used as the inoculum source. In the greenhouse, the cuttings of the mapping population were cultivated in a climatic chamber at 24 °C, young growing shoots with at least five fully expanded leaves were sprayed with conidia suspension adjusted to 10^4^ conidia/mL in the late afternoon and kept at 100% relative humidity overnight. In the field, clusters at susceptible phenological stage [26] BBCH 77 (berries beginning to touch) [89] and growing shoots were sprayed after the sunset to avoid direct UV irradiation. Plastic bags were used to wrap inoculated organs, ensuring a high humidity treatment overnight. Bags were removed the following early morning before sunrise. Overall, two experiments were conducted on clusters in the two growing seasons 2020 (FC1) and 2021 (FC2), while in 2022, disease progression did not occur due to unusually high temperatures during the night (>30 °C). In each field inoculation trial, the entire population was screened simultaneously, and two clusters were evaluated per genotype, generating four sub-experiments (FC1a, FC1b, FC2a, FC2b). Shoots were evaluated once in the field in 2021, distinguishing between leaves (FL) and shoot internodes (FS). A total of eight greenhouse inoculation trials were performed between 2020 and 2021 on subgroups of the population to obtain three experiments (GL1, GL2, GL3). To produce an overall dataset of the resistance trait, the replicated data of GL and FC trials were also combined by (i) retaining only the minimum resistance value per each genotype and experiment type (GL min and FC min), and (ii) calculating the median (GL median, FC median). While the minimum should exclude possible false positive resistance evaluations, the median is the most robust statistical parameter for non-normally distributed data. Resistance evaluation of leaves in the greenhouse was conducted following the 5-step scale proposed by Rex et al. [21], which resembles OIV descriptors [90], i.e., (1) very low, (3) low, (5) medium, (7) high, and (9) very high resistance. Contrariwise, leaves and shoot internodes in the field were evaluated with a binomial scale as susceptible (S that is 1) or resistant (R that is 9), respectively, in the presence or absence of lesions, because the occurrence of other lesions caused by multiple abiotic and biotic stresses did not permit using a more detailed scale. To screen cluster resistance in the field, a 5-step rating scheme was developed based on the percentage of infected berries (Table 3), following the example of OIV_459_ descriptor for degree of cluster resistance to Botrytis bunch rot [90]. Berry color (white or black, coded as a 0/1 valued binary variable) was also recorded to be used as a positive control in QTL analyses, thanks to the well-known location of the responsible QTL on chromosome 2 [33]. The normality of trait distribution was determined by means of the Shapiro–Wilk normality test (*p* < 0.05). All statistical analyses were executed with the software PAST 3.26 [91].

### 4.3. Genotyping

Genomic DNA extraction from young leaf tissue, quality evaluation and quantification, and array hybridization by Illumina SNPchip Infinium HD Ultra, GrapeReSeq 20K technology were performed at the Sequencing and Genotyping Platform of FEM (San Michele all’Adige, Italy). GenomeStudio 2.0 software (Illumina, USA) was used for SNP calling (GenCall score cutoff 0.15). A call rate of 95% was used to discard poorly performing samples. Quality control parameters as cluster separation score, call frequency, and measures of deviation from expected allele Mendelian inheritance patterns were used to identify SNPs that needed to be manually re-clustered or discarded [92]. The SNP scoring tool ASSiST 1.02 [93] was then implemented, with default thresholds, to filter the dataset and keep only robust SNPs segregating in the population. ASSiST was also used to recode the SNP dataset into JoinMap^®^ input format [31]. The SNP dataset was integrated with 192 SSR markers of the M × T map published by Vezzulli et al. [88]. Finally, three SSR markers that were previously associated with the *Rgb*1 locus [40] were amplified in triplex with KAPA2G Fast Multiplex Mix (Sigma-Aldrich/Merck, USA) and included in this study, namely GF14-42 [21], GF14-04 [94] and UDV-095 [41]. The amplification followed a touchdown protocol (60 to 55 °C, −0.5 °C × 10 cycles). Primer sequences and other details are available in (Table S10). The marker UDV-095 was already present in the map from Vezzulli et al. [88], and it served as internal control for SNP and SSR dataset integration. To be run in JoinMap^®^, the markers were renamed to have a maximum of 20 characters and to easily distinguish SSR from SNPs by a suffix (_R) (Table S11).

### 4.4. Linkage Mapping

High-density genetic maps were generated with the software JoinMap^®^ 5.0 [31] by determining LGs through independence LOD. Map quality improvements were achieved following the good practices for datasets obtained by the GrapeReSeq Illumina 20K SNPchip described in Vervalle et al. [95], i.e., chain length and stop criterion parameters optimization to allow convergence of the mapping algorithm. Firstly, an integrated map comprehending all robust SNPs and SSRs was calculated with Cross Pollination (CP) population type (wIN map). Being the number of loci per LG > 100, Maximum Likelihood algorithm was applied for map construction with the required Haldane’s mapping function. This analysis also produced parental M and T maps. The same pipeline was followed on a reduced dataset excluding identical segregating markers through the dedicated JoinMap function and producing rIN, rM and rT maps. Afterward, doubled-haploid maternal (dhM) and paternal (dhT) maps were calculated following the two-way pseudo-testcross strategy, [96], to overcome the problem of high number of dominant and missing observations of CP population, i.e., the memory limitations for QTL mapping [31]. To this purpose, the JoinMap function ‘create paternal and maternal map’ was used to extract loci segregating in each parent and transform them in biallelic markers, *lm* × *ll* and *nn* × *np* respectively. Loci of segregation type *hk* × *hk* were excluded from the dataset to avoid excess of missing data, as there is no possibility to reconstruct from which parent the alleles of heterozygote (*hk*) offspring derived [31]. Then, markers were converted in doubled-haploid segregation type *a* × *b*, with ‘*a*’ being either ‘*lm*’ or ‘*np*’ in the two sub-datasets, and identical loci were excluded. Due to the reduced number of loci, Regression mapping algorithm was also implemented and both Haldane’s and Kosambi’s mapping functions were tested to obtain the doubled-haploid maps. Since segregation distortion is a common phenomenon in outcrossing species as grapevine, its values were not used as a threshold to exclude markers [97]. Rather, the premature ungrouping—at lower LOD values compared to the other markers belonging to a linkage group—was used to exclude markers prior to grouping calculation. Other marker exclusion criteria applied to map construction were diagnostic values that stand out compared to the rest of the dataset, as high (i) nearest neighbor fit (NN Fit) and stress (NN Stress) that measure how well the markers fit that map order, and (ii) high -Log10(P) Genotype Probabilities Locus Means values, that measure how unlikely genotype observations affect the quality of the map [31,95]. Marker exclusion was checked through the function ‘combine maps’, to control if it corrupted the order of the map. The overall improvement in the quality of the maps was verified through the increasing (least negative value) of the logE-likelihood [98,99]. Maps were also combined to verify order consistency among complete, reduced and doubled-haploid integrated, maternal and paternal maps.

Linkage maps were visualized through the LinkageMapView package [32] using R [100] in the Rstudio environment [101], and MapChart [102].

### 4.5. QTL Analysis

The study of QTLs was performed with MapQTL^®^ 6.0 [103], and the two-way pseudo-testcross strategy [96] was employed to inspect the contribution of the resistant parent ‘Merzling’ to the trait. The KW non-parametric test (*p* < 0.005, ****) and Interval Mapping (IM, with Regression mapping algorithm) were implemented to highlight putative QTLs. Automatic cofactor selection (ACS) was then used to confirm significantly associated markers (*p* < 0.02). To this purpose, a set of cofactors selected at the constant distance of 1 cM on the chromosome where the putative QTL was detected was selected as starting set. This procedure was applied to allow the identification of multiple QTLs on the same chromosome, thus avoiding the occurrence of false positive ‘ghost QTL’ between adjacent QTLs [103]. The identified cofactors were then included in MQM with Regression mapping algorithm. For IM and MQM analyses, minimum GW LOD thresholds (*p* < 0.01, ^§§§§^; *p* < 0.05, ^§§§^) and CW (chromosome-wise)-LOD thresholds (*p* < 0.01, ^§§^; *p* < 0.05, ^§^) were calculated per each trait by three runs of 1000 permutation test, to determine four categories of QTL significance. Multiple rounds of MQM were run until no more loci exceeded CW^§^ thresholds. Adjacent significant loci commonly arose as output of the analysis, but they were not selected as cofactors following the general rule of one marker per locus. Quantitative trait loci were considered major QTLs if explaining more than 10% of the phenotypic variance, otherwise they were considered minor QTLs [34], and a confidence interval of two-LOD was applied to determine QTL limits. Finally, recombinant individuals in the surrounding QTL regions were inspected to deepen the genetic basis of the traits.

### 4.6. Candidate Gene Identification

The latest assembly version 4 of the PN40024 reference genome [35] was used in this work to define the genomic physical positions, since it properly replaced many genes thanks to the increased scaffold size—achieved by 30× PacBio data—assigning to the proper chromosome unknown contigs and genes belonging to heterozygous regions. For this reason, the flanking sequences of all SNPs and SSRs primers—retrieved from [104], and [21,41,88,94], respectively—were blasted on PN40024.v4 [105] to redefine their physical coordinates. Then, the new positions of the markers defining the two-LOD QTL confidence intervals were used to extract the list of genes located within the QTLs and extended to the nearest non-associated marker. This extension was intended to avoid losing associated genes, since it is not possible to determine with which marker the enclosed region co-segregates. Besides the genes annotated according to PN40024.v4.2 (V4.2) [106] two further gene lists were obtained through the Grapevine Expression Atlas web resource (GREAT, account mandatory) [107] to retrieve the V3 functional gene annotation [36], and the correspondence between V4 [106] and previous annotation i.e., CRIBI V1, CRIBI V2, and Vcost V3 [38]. Finally, the lists were merged, and the QTL regions inspected using the genome browser interface Jbrowse [108]. If new genes were predicted in V4, they were retained in the list, while if the V4 lacked genes from other annotation versions, their localization was checked to determine whether to exclude them. The candidate gene lists were studied through GO enrichment executed through ShinyGO 0.76.3 [109] with false discovery rate (FDR) cutoff of 0.05 and by selecting ‘All available gene sets’ as pathway database. Basic local alignments were performed at the PN40024.v4 [35] dedicated BLAST sequence server [105]. Multiple sequence alignments were implemented at MUSCLE [110] and the visualization realized through the linked software Mview© 1.63 [111]. Protein sequence conservation was visualized through the NCBI web application Multiple Sequence Alignment Viewer 1.22.2 [112] with MUSCLE alignment as input (Pearson/FASTA format) and ‘Column Quality Score’ coloring method, that assigns scores to amino acids based on how well they agree with the others at that position. The phylogenetic reconstructions were performed with the web application CLUSTALW [113] using the function ‘FastTree’ [114] to generate phylograms, that are phylogenetic trees that have branch lengths proportional to the amount of character change [115]. FastTree v2.1.8 function [114] uses a non-parametric branch support based on a Shimodaira–Hasegawa procedure (SH-like) [116] to provide local support SH-like values, that represent the degree of genetic change ranging from 0 to 1. Divergences were verified at structural annotation level through the platform Apollo annotator (training and account mandatory) [117], and manually curated if necessary. In specific cases, functional annotation was further inspected at various databases e.g., InterPro [118], TAIR [119] or UniProt [120] databases. Gene expression was also explored exploiting published data for the susceptible *V. vinifera* ‘Corvina’ at different developmental stages [39] by using the Expression Atlases App (EX-ATLAS) [121], provided by TOMSBio Lab Vitis Visualization (VITVIZ) platform [122].

## 5. Conclusions

The novel QTL associated with BR bunch resistance, together with the confirmed QTL linked to shoot resistance, has the potential to become a resource in different frameworks. Firstly, general hypotheses regarding resistance mechanisms were highlighted and set the stage for future basic research on putative susceptibility genes, the Germin-like protein cluster, and metabolites involved in mitochondrial oxidative burst and phloem occlusion. To unravel this knot of information, the Telomere–to–Telomere (T2T) sequencing of the resistance donor represents the first step and is envisaged. Secondly, this study represents a breakthrough in applied research towards marker-assisted and genomics-aided breeding for BR resistance. In fact, the deployment of organ-specific QTLs is desirable to contain primary infection on leaves and to protect bunches from secondary infection. To reach the goal of routinary adoption, the validation step of the associated markers is essential and already planned in segregating populations derived from different genetic backgrounds. Finally, exploring diverse sources of resistance is crucial to prevent overcoming by distinct pathogen strains and ensure BR resistance durability.

## Figures and Tables

**Table 1:** Summary of overall map characteristics: total genetic length, marker number in the whole and reduced datasets, number and density of unique bins, inter locus mean distance, and maximum gap size across the integrated and parental linkage maps of the M × T population. Abbreviations: cM = centimorgan; dh = doubled haploid; IN = integrated; LG = linkage group; M = ‘Merzling’; T = ‘Teroldego’.

Map	Length	Markers		Bins			Max Gap	
		Whole	Reduced	N°	Density ^a^	Mean Distance ^b^	cM	LG
IN	1677	7358	3249	1613	0.96	1.04	9.5	15
M	1452	4681	2218	849	0.58	1.71	15.7	12
T	1743	4553	2348	971	0.56	1.80	28.0	18
dhM	943	2884	998	791	0.84	1.19	13.2	6
dhT	1346	2744	1137	917	0.68	1.47	22.0	18

^a^ density = bin n°/map length. ^b^ mean distance = map length/bins n°.

**Table 2:** Description of the significant (*p* < 0.01) QTLs on LG14 associated with black rot resistance identified in ‘Merzling’. When a blank space is given, it means that the value was equal to that of the cell above it. Abbreviations: cM = centimorgan; FC = field cluster; FL = field leaf; FS = field shoot internode; FC = field cluster; GL = greenhouse leaf; LG = linkage group; LOD = logarithm of odds; min = minimum; PVE = phenotypic variance explained.

QTL	Organ	Trial	Most Associated Marker	LOD	Two-LOD Interval	Length (cM)	PVE	Additive Effect
*Rgb*1	Leaf	GL1	GF14-42	8.47	40.584–40.626	0.04	29.2%	1.32
		GL2		4.86			20.4%	1.36
		GL3		7.13			22.9%	1.28
		GL min		5.79			18.4%	1.01
		FL		7.59			26.2%	1.83
	Shoot internode	FS		6.63			26.3%	2.03
*Rgb*3	Cluster	FC1a	C14_20097630ae	3.66	31.241–31.533	0.29	22.5%	1.24
		FC1b		2.22			15.7%	0.94
		FC1		5.96			20.3%	1.22
		FC2a		27.63			75.7%	3.20
		FC2b		35.70			83.9%	3.42
		FC2		31.38			79.9%	3.36
		FC min		24.34			68.9%	3.10

**Table 3:** Rating scale used for the evaluation of degree of cluster resistance to black rot.

Rate	Description
1	very low resistant: more than 60% of infected berries with pycnidia formation
3	low resistant: up to 60% of infected berries with pycnidia formation
5	medium resistant: no more than 20% infected berries with pycnidia formation
7	high resistant: few infected berries with pycnidia formation
9	very high resistant: no infected berries

## Data Availability

The data presented in this study are available in Supplementary Materials.

## Supplementary Materials

The following supporting information can be downloaded at: https://www.mdpi.com/article/10.3390/ijms24043568/s1.
