# Supplementary material for: Towards Marker-Assisted Breeding for Black Rot Bunch Resistance: Identification of a Major QTL in the Grapevine Cultivar ‘Merzling’
Source: Int J Mol Sci. 2023 Feb 10;24(4):3568. doi: 10.3390/ijms24043568 (PMC9961920; doi:10.3390/ijms24043568)
Supplement: Supplementary file 1 [file ijms-24-03568-s001.zip › Figure S4.pdf]

```
Reference sequence (1): Vitvi14g02865
Identities normalised by aligned length.
Colored by: identity

                     cov    pid   1 [        .         .         .         .         :         .         .         . 80
 1 Vitvi14g02865  100.0% 100.0%     -------------MKNMVINTLACIALLAMSFFLASVSDPSPLQEFCVAVNDTKTTVFVNGKVCKDLKVATANDLFFSRL
 2 Vitvi14g01108   92.1%  75.6%     -------------MESILVKHRTPLP-PALPISLA----------FTPRTFGTIALLFVNGKVCKDPKVATANDFFFSGL
 3 NA1             79.4%  72.4%     -------------MLQL----------------------------------------FVNGKVCKDPKVATANDFFFSGL
 4 Vitvi14g01106   80.7%  73.8%     -------------M-------LQC------------------------------ERVFVNGKVCKDPKVATANDFFFSGL
 5 Vitvi14g01080   96.9%  84.6%     MSMSSFRWAIALRCIRRGINTLACIALLAMSFFLASASDPSPLQDFCVAVNDTKTTVFVNGKVCKDPKVATANDFFFSGL
 6 Vitvi14g01075   96.9%  89.6%     -------------MKKMVVNTLASIPLLAMSFFLAFASDPSPLQDFCVAVNDTKTTVFVNGKVCKDPKVATPNDFFFSGL
 7 Vitvi14g01113   96.9%  90.0%     -------------MKKMVVNTLACIALSAMSFFLASASDPSPLQDFCVAVNDTKTTVFVNGKVCKDPKVATADDFFFSGL
 8 Vitvi14g01082   96.9%  90.5%     -------------MKKMVVNTLACIALSAMSFFLASASDPSPLQDFCVAVNDTKTTVFVNGKVCKDPKVATANDFFFSGL
 9 Vitvi14g04720   96.9%  92.3%     -------------MKKMVINTLACIALLAMSFFLASASDPSPLQDFCVAVNDTKTTVFVNGKVCKDPKVATANDFFFSGL
10 Vitvi14g01096   96.9%  91.9%     -------------MKKMVINTLACIALSAMSFFLASASDPSPLQDFCVAVNDTKTTVFVNGKVCKDPKVATANDFFFSGL
11 NA2             96.9%  91.9%     -------------MKKMVINTLACIALLAMSFFLASASDPSPLQDFCVAVNDTKTTVFVNGKVCKDPKVATANDFFFSGL
12 Vitvi14g04724   96.9%  91.9%     -------------MKKMVINTLACIALLAMSFFLASASDPSPLQDFCVAVNDTKTTVFVNGKVCKDPKVATANDFFFSGL
13 Vitvi14g01112   96.9%  91.9%     -------------MKKMVINTLACIALLAMSFFLASASDPSPLQDFCVAVNDTKTTVFVNGKVCKDPKVATANDFFFSGL
14 Vitvi14g02863   96.9%  91.4%     -------------MKKMVINTLACIALSAMSFFLASASDPSPLQDFCVAVNDTKTTVFVNGKVCKDPKVATANDFFFSGL
15 Vitvi14g02864   96.9%  91.4%     -------------MKKMVINTLACIALSAMSFFLASASDPSPLQDFCVAVNDTKTTVFVNGKVCKDPKVATANDFFFSGL
   consensus/100%                   .............h...........................................FVNGKVCKD.KVATssDhFFStL
   consensus/90%                    .............M.ph....hts..............................thlFVNGKVCKDPKVATANDFFFSGL
   consensus/80%                    .............McphllNTLACIsL.AMSFFLA.sSDPSPLQ-FCVAVNDTKTTVFVNGKVCKDPKVATANDFFFSGL
   consensus/70%                    .............MK+MVlNTLACIAL.AMSFFLASASDPSPLQDFCVAVNDTKTTVFVNGKVCKDPKVATANDFFFSGL

                     cov    pid  81          .         1         .         .         .         .         :         . 160
 1 Vitvi14g02865  100.0% 100.0%     QVLGHTSNKLESMVTQANVAQIPELNTLGISLARVDYAPYGLNPPHTYPRATEILTVLEGTLYVGFVTSNPDNRLISKVL
 2 Vitvi14g01108   92.1%  75.6%     RVPGNTSNKLGSMVTPANVAQIPDLKTLGISLARVDYAPYGLNPPHTHPRATEILTVLEGTLYVGFVTSNPDNRLISKVL
 3 NA1             79.4%  72.4%     RVPGNTSNKLGSMVTPANVAQIPDLKTLGISLARVDYAPYGLNPPHTHPRDTEILTFLEGTLYVGFVTSNPDNRLISKVL
 4 Vitvi14g01106   80.7%  73.8%     RVPGNTSNKLGSMVTPANVAQIPDLKTLGISLARVDYAPYGLNPPHTHPRDTEILTFLEGTLYVGFVTSNPDNRLISKVL
 5 Vitvi14g01080   96.9%  84.6%     RVPGNTSNKLGSMVTPANVAQIPGLNTLGISLARVDYAPYGLNPPHTHPRATEILTVLEGTLYVGFVTSNPDNRLIAKVL
 6 Vitvi14g01075   96.9%  89.6%     RVPGNTSNKVGSMVTPANVAQIPGLNTLGISLARVDYAPYGLNPPHTHPRATEILTVLEGTLYVGFVTSNPDNRLISKVL
 7 Vitvi14g01113   96.9%  90.0%     RVPGNTSNKVGSMVTPANVAQIPGLNTLGISLARVDYAPYGLNPPHTHPRATEILTVLEGTLYVGFVTSNPDNRLICKVL
 8 Vitvi14g01082   96.9%  90.5%     RVPGNTSNKVGSMVTPANVAQIPGLNTLGISLARVDYAPYGLNPPHTHPRATEILTVLEGTLYVGFVTSNPDNRLICKVL
 9 Vitvi14g04720   96.9%  92.3%     RVPGNTSNKLGSMVTPANVAQIPGLNTLGISLARVDYAPYGLNPPHTHPRATEILTVLEGTLYVGFVTSNPDNRLISKVL
10 Vitvi14g01096   96.9%  91.9%     RVPGNTSNKLGSMVTPANVAQIPGLNTLGISLARVDYAPYGLNPPHTHPRATEILTVLEGTLYVGFVTSNPDNRLISKVL
11 NA2             96.9%  91.9%     RVPGNTSNKVGSMVTPANVAQIPGLNTLGISLARVDYAPYGLNPPHTHPRATEILTVLEGTLYVGFVTSNPDNRLISKVL
12 Vitvi14g04724   96.9%  91.9%     RVPGNTSNKVGSMVTPANVAQIPGLNTLGISLARVDYAPYGLNPPHTHPRATEILTVLEGTLYVGFVTSNPDNRLISKVL
13 Vitvi14g01112   96.9%  91.9%     RVPGNTSNKVGSMVTPANVAQIPGLNTLGISLARVDYAPYGLNPPHTHPRATEILTVLEGTLYVGFVTSNPDNRLISKVL
14 Vitvi14g02863   96.9%  91.4%     RVPGNTSNKVGSMVTPANVAQIPGLNTLGISLARVDYAPYGLNPPHTHPRATEILTVLEGTLYVGFVTSNPDNRLISKVL
15 Vitvi14g02864   96.9%  91.4%     RVPGNTSNKVGSMVTPANVAQIPGLNTLGISLARVDYAPYGLNPPHTHPRATEILTVLEGTLYVGFVTSNPDNRLISKVL
   consensus/100%                   pV.GpTSNKltSMVT.ANVAQIPtLpTLGISLARVDYAPYGLNPPHTaPRsTEILThLEGTLYVGFVTSNPDNRLIsKVL
   consensus/90%                    RVPGNTSNKlGSMVTPANVAQIPsLpTLGISLARVDYAPYGLNPPHTHPRsTEILThLEGTLYVGFVTSNPDNRLIsKVL
   consensus/80%                    RVPGNTSNKlGSMVTPANVAQIPsLNTLGISLARVDYAPYGLNPPHTHPRATEILTVLEGTLYVGFVTSNPDNRLISKVL
   consensus/70%                    RVPGNTSNKlGSMVTPANVAQIPGLNTLGISLARVDYAPYGLNPPHTHPRATEILTVLEGTLYVGFVTSNPDNRLISKVL

                     cov    pid 161          .         .         .         2         .         .         .         . 240
 1 Vitvi14g02865  100.0% 100.0%     YKGDVFIFLEGLIHFQLNVRKTKAVAIAALSSQNLGVITIANAVFGSKPAISADVLTKAFQVDKKVVDYLQSQFWTDNHT
 2 Vitvi14g01108   92.1%  75.6%     YKGDVFVFPEDLIHFQLNVEKTKAVAIAALSSQNPGVITIANAVFGSKPAISADVLTKAFQVDKKVVDYLQSQF------
 3 NA1             79.4%  72.4%     YKGDVFVFPEDLIHFQLNVEKTKAVAIAALSSQNPGVITIANAVFGSKPAISADVLTKAFQVDKKVVDYLQSQF------
 4 Vitvi14g01106   80.7%  73.8%     YKGDVFVFPEDLIHFQLNVEKTKAVAIAALSSQNPGVITIANAVFGSKPAISADVLTKAFQVDKKVVDYLQSQF------
 5 Vitvi14g01080   96.9%  84.6%     YKGDVFVFPEGLIHFHLNVGKTKAVAIAALSSQNPGVITIANAVFGSKPAISADVLTKAFQVDKKVVDYLQSQF------
 6 Vitvi14g01075   96.9%  89.6%     YKGDVFVFPEGLIHFQLNVGKTKAVAIAALSSQNPGVITIANAVFGSKPAISADVLTKAFQVDKKVVDYLQSQF------
 7 Vitvi14g01113   96.9%  90.0%     YKGDVFIFPEGLIHFQLNVGKTKAVAIAALSSQNPGVITIANAVFGSKPAISADVLTKAFQVDKNVVDYLQSQF------
 8 Vitvi14g01082   96.9%  90.5%     YKGDVFVFPEGLIHFQLNVGKTKAVAIAALSSQNPGVITIANAVFGSKPAISADVLTKAFQVDKKVVDYLQSQF------
 9 Vitvi14g04720   96.9%  92.3%     YKGDVFVFPEGLIHFQLNVGKTKAVAIAALSSQNPGVITIANAVFGSKPAISADVLTKAFQVDKKVVDYLQSQF------
10 Vitvi14g01096   96.9%  91.9%     YKGDVFVFPEGLIHFQLNVGKTKAVAIAALSSQNPGVITIANAVFGSKPAISADVLTKAFQVDKKVVDYLQSQF------
11 NA2             96.9%  91.9%     YKGDVFVFPEGLIHFQLNVGKTKAVAIAALSSQNPGVITIANAVFGSKPAISADVLTKAFQVDKKVVDYLQSQF------
12 Vitvi14g04724   96.9%  91.9%     YKGDVFVFPEGLIHFQLNVGKTKAVAIAALSSQNPGVITIANAVFGSKPAISADVLTKAFQVDKKVVDYLQSQF------
13 Vitvi14g01112   96.9%  91.9%     YKGDVFVFPEGLIHFQLNVGKTKAVAIAALSSQNPGVITIANAVFGSKPAISADVLTKAFQVDKKVVDYLQSQF------
14 Vitvi14g02863   96.9%  91.4%     YKGDVFVFPEGLIHFQLNVGKTKAVAIAALSSQNPGVITIANAVFGSKPAISADVLTKAFQVDKKVVDYLQSQF------
15 Vitvi14g02864   96.9%  91.4%     YKGDVFVFPEGLIHFQLNVGKTKAVAIAALSSQNPGVITIANAVFGSKPAISADVLTKAFQVDKKVVDYLQSQF------
   consensus/100%                   YKGDVFlF.EsLIHFpLNVtKTKAVAIAALSSQN.GVITIANAVFGSKPAISADVLTKAFQVDKpVVDYLQSQF......
   consensus/90%                    YKGDVFlFPEsLIHFQLNVtKTKAVAIAALSSQNPGVITIANAVFGSKPAISADVLTKAFQVDKKVVDYLQSQF......
   consensus/80%                    YKGDVFVFPEGLIHFQLNVtKTKAVAIAALSSQNPGVITIANAVFGSKPAISADVLTKAFQVDKKVVDYLQSQF......
   consensus/70%                    YKGDVFVFPEGLIHFQLNVGKTKAVAIAALSSQNPGVITIANAVFGSKPAISADVLTKAFQVDKKVVDYLQSQF......

                     cov    pid 241 ] 241
 1 Vitvi14g02865  100.0% 100.0%     N
 2 Vitvi14g01108   92.1%  75.6%     -
 3 NA1             79.4%  72.4%     -
 4 Vitvi14g01106   80.7%  73.8%     -
 5 Vitvi14g01080   96.9%  84.6%     -
 6 Vitvi14g01075   96.9%  89.6%     -
 7 Vitvi14g01113   96.9%  90.0%     -
 8 Vitvi14g01082   96.9%  90.5%     -
 9 Vitvi14g04720   96.9%  92.3%     -
10 Vitvi14g01096   96.9%  91.9%     -
11 NA2             96.9%  91.9%     -
12 Vitvi14g04724   96.9%  91.9%     -
13 Vitvi14g01112   96.9%  91.9%     -
14 Vitvi14g02863   96.9%  91.4%     -
```

```
 15 Vitvi14g02864   96.9%  91.4%     -
    consensus/100%                   .
    consensus/90%                    .
    consensus/80%                    .
    consensus/70%                    .
```

MView 1.63, Copyright © 1997-2018 Nigel P. Brown

Consensus pattern definitions

Consensus patterns are based on equivalence classes, that is, sets of residues that share some predefined property. These classes are not mutually exclusive and the consensus mechanism will choose the most specific class that summarizes a given column at the desired percent identity.

The default for protein alignments is called `P1` and is defined by physicochemical property as follows:

```
[P1]
#Protein consensus: conserved physicochemical classes, derived from
#the Venn diagrams of: Taylor W. R. (1986). The classification of amino
acid
#conservation. J. Theor. Biol. 119:205-218.
#description =>  symbol  members
*            =>  .
A            =>  A       { A }
C            =>  C       { C }
D            =>  D       { D }
E            =>  E       { E }
F            =>  F       { F }
G            =>  G       { G }
H            =>  H       { H }
I            =>  I       { I }
K            =>  K       { K }
L            =>  L       { L }
M            =>  M       { M }
N            =>  N       { N }
P            =>  P       { P }
Q            =>  Q       { Q }
R            =>  R       { R }
S            =>  S       { S }
T            =>  T       { T }
V            =>  V       { V }
W            =>  W       { W }
Y            =>  Y       { Y }
alcohol      =>  o       { S, T }
aliphatic    =>  l       { I, L, V }
aromatic     =>  a       { F, H, W, Y }
charged      =>  c       { D, E, H, K, R }
hydrophobic  =>  h       { A, C, F, G, H, I, K, L, M, R, T, V, W, Y }
negative     =>  -       { D, E }
polar        =>  p       { C, D, E, H, K, N, Q, R, S, T }
positive     =>  +       { H, K, R }
small        =>  s       { A, C, D, G, N, P, S, T, V }
tiny         =>  u       { A, G, S }
turnlike     =>  t       { A, C, D, E, G, H, K, N, Q, R, S, T }
```

Percent Identity Matrix - created by Clustal2.1

		1	2	3	4	5	6	7	8	9	10	11	12	13	14	Protein lenght
1	Vitvi14g02865															228
2	Vitvi14g01108	79.52														210
3	NA1	88.4	97.24													181
4	Vitvi14g01106	88.59	95.65	100												184
5	Vitvi14g01080	89.59	83.81	93.37	93.48											234
6	Vitvi14g01075	89.59	85.24	93.92	93.48	94.12										221
7	Vitvi14g01113	90.05	83.33	92.27	92.39	94.12	95.93									221
8	Vitvi14g01082	90.5	84.76	93.92	94.02	95.48	97.29	98.64								221
9	Vitvi14g04720	92.31	85.24	95.03	95.11	96.83	97.29	96.83	98.19							221
10	Vitvi14g01096	91.86	85.24	95.03	95.11	96.38	96.83	97.29	98.64	99.55						221
11	NA2	91.86	84.76	94.48	94.57	96.38	97.74	97.29	98.64	99.55	99.1					221
12	Vitvi14g04724	91.86	84.76	94.48	94.57	96.38	97.74	97.29	98.64	99.55	99.1	100				221
13	Vitvi14g01112	91.86	84.76	94.48	94.57	96.38	97.74	97.29	98.64	99.55	99.1	100	100			221
14	Vitvi14g02863	91.4	84.76	94.48	94.57	95.93	97.29	97.74	99.1	99.1	99.55	99.55	99.55	99.55		221
15	Vitvi14g02864	91.4	84.76	94.48	94.57	95.93	97.29	97.74	99.1	99.1	99.55	99.55	99.55	99.55	100	221
