# Supplementary figures and images for: Towards Marker-Assisted Breeding for Black Rot Bunch Resistance: Identification of a Major QTL in the Grapevine Cultivar ‘Merzling’
Source: Int J Mol Sci. 2023 Feb 10;24(4):3568. doi: 10.3390/ijms24043568 (PMC9961920; doi:10.3390/ijms24043568)

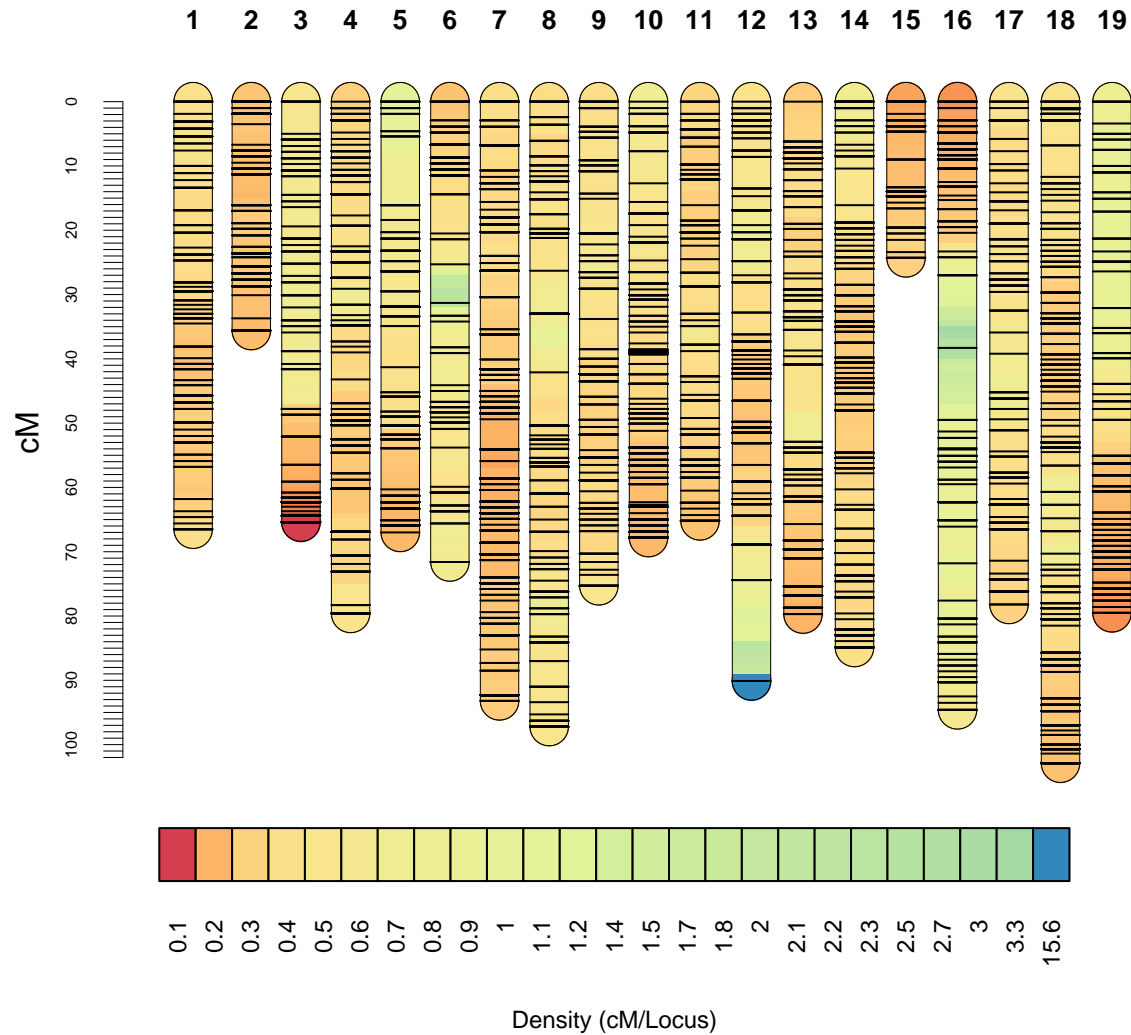

Supplement: Supplementary file 1 [file ijms-24-03568-s001.zip › Figure S2.pdf]

1 2 3 4 5 6 7 8 9 10 11 12 13 14 15 16 17 18 19

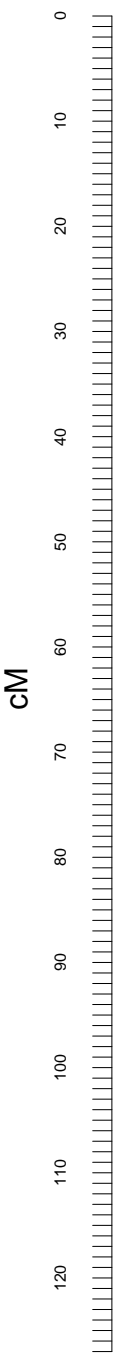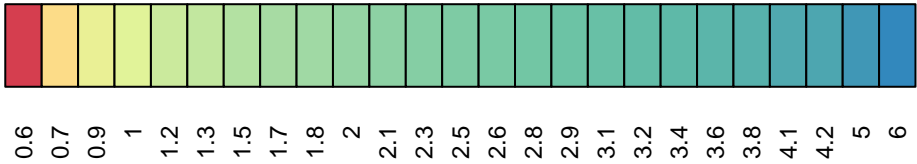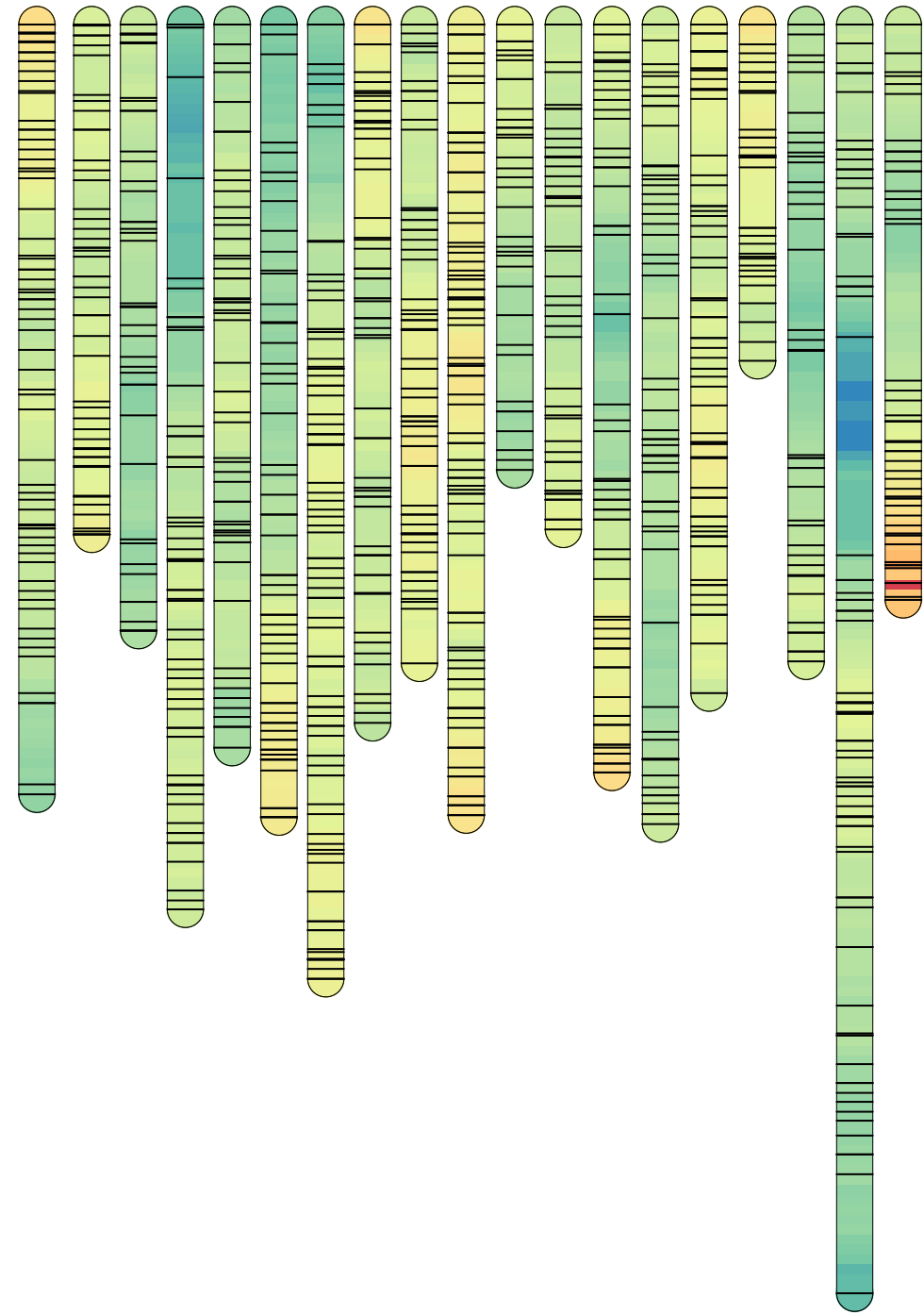

Supplement: Supplementary file 1 [file ijms-24-03568-s001.zip › Figure S3.pdf]

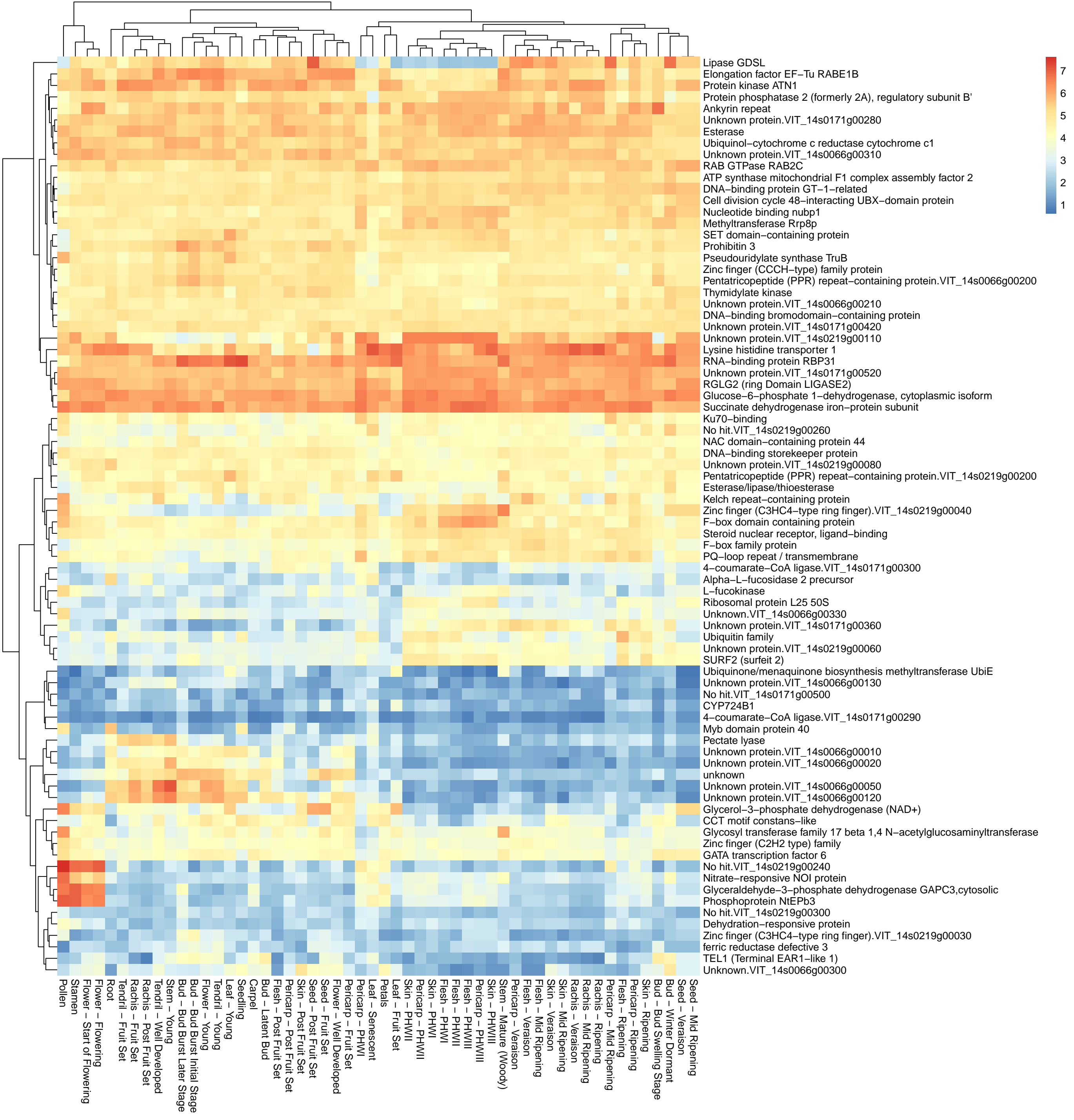

Supplement: Supplementary file 1 [file ijms-24-03568-s001.zip › Figure S5.pdf]
