# Supplementary material for: Towards Marker-Assisted Breeding for Black Rot Bunch Resistance: Identification of a Major QTL in the Grapevine Cultivar ‘Merzling’
Source: Int J Mol Sci. 2023 Feb 10;24(4):3568. doi: 10.3390/ijms24043568 (PMC9961920; doi:10.3390/ijms24043568)
Supplement: Supplementary file 1 [file Figure S1.pdf › Figure S1.pdf]

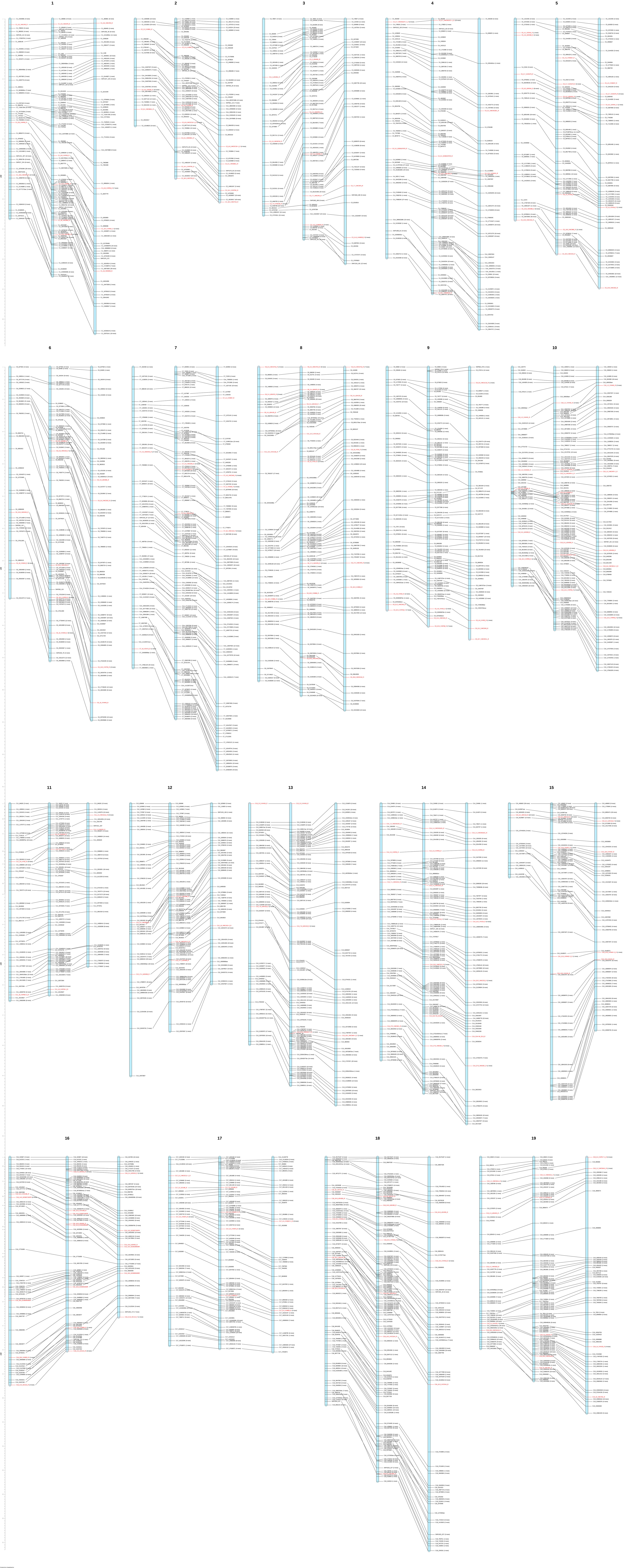
1
2
3
4
5
6
7
8
9
10
11
12
13
14
15
16
17
18
19
